# Editorial: Wild Plants as Source of New Crops

**DOI:** 10.3389/fpls.2020.591554

**Published:** 2020-09-11

**Authors:** Eric von Wettberg, Thomas M. Davis, Petr Smýkal

**Affiliations:** ^1^ Plant and Soil Science and Gund Institute for the Environment, University of Vermont, Burlington, VT, United States; ^2^ Department of Agriculture, Nutrition, and Food Systems, University of New Hampshire, Durham, NH, United States; ^3^ Department of Botany, Faculty of Science, Palacky University, Olomouc, Czechia

**Keywords:** crop wild relatives, domestication, genetic diversity, neo-domestication, breeding, ethnobotany

The history of agriculture can be viewed as a series of key events, such as the Neolithic Revolution, post-domestication expansion of agriculture to new regions, secondary domestications of new crops, movement over the Silk Road, the Columbian Exchange, the Industrial Revolution, the Green Revolution and even the more recent, ongoing genomic revolutions. Each of these has had positive benefits, but they have also come at a cost, including to agricultural biodiversity.

It is estimated that on the Earth there are between 300,000 and 500,000 species of higher plants, of which approximately 369,000 have been identified or described (Willis, 2017). Many species are still unknown to science, while perhaps a third is at risk of extinction (Pimm and Joppa, 2015) . The number of plant species used for food by pre-agricultural human societies is estimated to be around 7,000, but only a small fraction of the diversity of the plant kingdom has been domesticated. Our present knowledge of domesticated plants largely reflects our experience of a relatively small number of living domesticates adapted to recent, Holocene environments. The process of crop domestication was based on selection driven by human cultivation practices and agricultural environments. Approximately, 2,500 species have undergone some degree of domestication, and 250 species are considered to be fully domesticated, in the sense that their full lifecycle became dependent on human cultivation (Meyer et al., 2012; Gaut et al., 2018; Smýkal et al., 2018). Humanity relies on a small collection of crop plants such as corn, rice, wheat, soybean, and potato constituting the majority of our dietary intake. Altogether, some 10 to 50 plant species together provide about 95% of the world’s caloric intake. This concentration on a few species for most food is a key element of the vulnerability of the world food supply to the impact of climate change and the outbreak of major new plant diseases.

Crop wild relatives (CWRs) remain the largest reservoir of genetic diversity for crop improvement and have been utilized for major gene disease and pest resistance, and abiotic stress tolerance (Vavilov et al., 1992; Hajjar and Hodgkin, 2007; Warschefsky et al., 2014; Castañeda-Álvarez et al., 2016; Smýkal et al., 2018; Coyne et al., 2020). However, there remains a large set of plant species from various plant families and genera which have favorable traits but have not been domesticated so far. As we have been gaining knowledge on the genomic and biological background of domestication processes, we can apply more effective selection to domesticate more wild species. Since many wild taxa are locally adapted to particular habitats and contain significant genetic diversity, this might create novel crops and help us to achieve more environmentally sustainable agriculture as we face climate change. Not all candidates for neo-domestication are CWR, although many and perhaps most will be, because the form/function of the related crop species provides a useful template to guide the neo-domestication of the CWR. On the other hand, all of the wild sources of useful genes for introgression into crop species are necessarily CWR as a prerequisite for their ability to cross with a crop relative. So, a given CWR can be a valuable source for gene introgression into its crop relative, and can at the same time be a candidate for neo-domestication.

Given our reliance on fewer and fewer crop species, as well as the need to more sustainably intensify agricultural production and to develop more nutritious crops, utilizing a greater range of plant diversity is critically important. While conventional breeding by hybridization and progeny selection augmented by marker-assisted selection are likely to be the most practicable approaches for the time-being, recent developments in genome editing technologies, genomic selection, and inoculation of plants with beneficial microbes may help accelerate this process in the future by converting the wild forms of key domestication genes to their respective domesticated forms, as reviewed by Van Tassel et al.

Several tools exist for those wishing to neo-domesticate a wild species or to make an existing crop more tractable to genetic improvement. The most obvious of newly emerging tools are gene editing approaches. Although not technically trivial, gene editing offers a neo-domesticator the opportunity to simply engineer the domestication traits of interest, such as indehiscent propagules, based on knowledge from other taxa (e.g., Ogutcen et al., 2018). However, other tools of modern genomics may be useful to those wishing to neo-domesticate a crop. These include genome sequencing and resequencing to characterize molecular variation, Genome Wide Association Studies (GWAS) to find associations between genes and traits of interest and genomic selection to improve multi-genic traits in a breeding program. We also review several core concepts in neo-domestication. Our special issue focuses nearly equally on neodomestication of wild species and harnessing an understanding of domestication traits to improve existing crop species.

This Research Topic, Wild Plants as Source of New Crops, covers the following topics:

1. De Novo Domestication of Plant Species
  One of our examples of *de novo* domestication comes from Takahashi et al., who examine neo-domestication in *Vigna stipulaceae*. The genus *Vigna*, which includes nine independent domestications, is particularly ripe for domestication. Many of the wild species, such as *V. stipulaceae*, have useful adaptation traits such as drought tolerance or salinity tolerance, making them desirable targets for neo-domestication. Renzi et al. have looked at *Vicia villosa*, a stress tolerant and rapidly growing forage. Kissing Kucek et al. addressed the shattering pod traits that makes *V. villosa* weedy and difficult to control in field settings. Selecting for non-dehiscence would help to domesticate this useful species. Common vetch has also been utilized as a drought tolerant forage (Nguyen et al.). Another take on neo-domestication is to look for traits that may pre-adapt certain lineages to domestication. Bronnvik and von Wettberg look at bird dispersal in legumes as a possible trait preadapting some legumes toward indehiscent pods.
2. Use of Crop Wild Relatives to Broaden Genetic Diversity of Crops
  Crop wild relatives are an important source of variability for existing crops and can be used more effectively when the genetic basis of domestication traits are understood. Henry reviews the diversity of wild rice relatives in Australia, while Wang et al. use GWAS analysis of weedy rice in relation to seed nutritional quality. Berny Mier y Teran et al. have examined wild relatives of common bean, *Phaseolus vulgaris*, with introgression populations. Ivanizs et al. examine the potential of *Aegilops biuncialis* for wheat improvement. Sharma et al. examine the tertiary genepool of pigeonpea, *Cajanus cajan*. Interesting re-discovery of two coffee species is reported by Davis et al.
3. Use of Perennial Species
Crain et al. have used genomic selection for improvement of intermediate wheatgrass. Herron et al. examine early growth traits across the genus *Phaseolus*, with an emphasis on wild perennial taxa, with the aim of identifying perennial species that might be suitable targets for neo-domestication. An intriguing illustration of domestication parallels is provided by analysis of annual and perennial sunflowers (Asselin et al.). There are many domesticated tree species. They differ from typical seed propagated annual or perennial species due to their long lifespan and combination of sexual and clonal propagation. Differences in seed anatomy and germination capacity of wild and cultivated passion fruit have been studied by Castillo et al.
4. Novel Domestication Syndromes
Zhao et al. have examined the domestication of *Zizania latifolia*, a grass with a novel domestication as a vegetable, where a symbiosis with an endosymbiotic fungus is essential to the domestication syndrome.
5. Medicinal and Horticultural Plants, Their Domestication, and Diversity
Pasta et al. have examined the flora of Sicily for edible plants, in an effort to preserve ecological knowledge and potential medicinal value. Letelier et al. make a similar examination of the flora of Chile. A genomic approach to chromosome scale assembly of garden orach (*Atriplex hortensis*) is presented by Hunt et al. The use of CWR germplasm for cultivar improvement of the widely used herb mint (*Mentha*) is shown by Vining et al.

## Author Contributions

TD, EW, and PS have equally contributed to writing and final editing. All authors contributed to the article and approved the submitted version.

## Funding

PS is supported by the Grant Agency of the Czech Republic and Palacký University grant Agency [IGA-2020_003]. EW is supported by the USDA Hatch program through the Vermont State Agricultural Experimental Station and is supported to work on diversity of mungbeans by Russian Scientific Fund Project No. 18-46-08001 on the basis of a unique scientific installation “Collectionof plant genetic resources VIR”. TD is supported in part by the USDA National Institute of Food and Agriculture Hatch Project NH00678 (accession number 1019990).

## Conflict of Interest

The authors declare that the research was conducted in the absence of any commercial or financial relationships that could be construed as a potential conflict of interest.
