# Corrigendum: Editorial: Wild Plants as Source of New Crops

**DOI:** 10.3389/fpls.2020.638134

**Published:** 2021-01-14

**Authors:** Eric von Wettberg, Thomas M. Davis, Petr Smýkal

**Affiliations:** ^1^Plant and Soil Science and Gund Institute for the Environment, University of Vermont, Burlington, VT, United States; ^2^Institute for Applied Mathematics and Mechanics, Peter the Great St. Petersburg Polytechnic University, St. Petersburg, Russia; ^3^Department of Agriculture, Nutrition, and Food Systems, University of New Hampshire, Durham, NH, United States; ^4^Department of Botany, Faculty of Science, Palacky University, Olomouc, Czechia

**Keywords:** crop wild relatives, domestication, genetic diversity, neo-domestication, breeding, ethnobotany

In the published article, there was an error regarding the affiliation**(s)** for **Eric von Wettberg**. As well as having affiliation(s) **1**, they should also have **2**.

Additionally, in the original article, we neglected to include the funder **USDA National Institute of Food and Agriculture Hatch Project**, **NH00678** to Thomas M. Davis. A correction has therefore been made to the **Funding s**tatement.

**“**PS is supported by the Grant Agency of the Czech Republic and Palacký University grant Agency [IGA-2020_003]. EW is supported by the USDA Hatch program through the Vermont State Agricultural Experimental Station and is supported to work on diversity of mungbeans by Russian Scientific Fund Project No. 18-46-08001 on the basis of a unique scientific installation “Collection of plant genetic resources VIR”. TD is supported in part by the USDA National Institute of Food and Agriculture Hatch Project NH00678 (accession number 1019990).”

The authors apologize for these errors and state that this does not change the scientific conclusions of the article in any way. The original article has been updated.

